# Mental health apps within the healthcare system: associations with stigma and mental health literacy

**DOI:** 10.1186/s13690-024-01362-w

**Published:** 2024-08-16

**Authors:** Sophia Fürtjes, Mariam Al-Assad, Hanna Kische, Katja Beesdo-Baum

**Affiliations:** Behavioral Epidemiology, Institute of Clinical Psychology and Psychotherapy, Technische Universität Dresden, Chemnitzer Straße 46, Dresden, 01187 Germany

**Keywords:** Mental health apps, Service utilization, Stigma, Healthcare system

## Abstract

**Background:**

Mental health apps (MHA) as a new form of self-help have gained popularity over the last years. Tentative evidence has suggested that MHA might also present a first step into the help-seeking process, because their anonymity circumvents stigma. Using MHA might also increase mental health literacy and reduce stigma through psychoeducation, which could encourage formal help-seeking. To date, it remains unclear how MHA usage relates to stigma, mental health literacy, and utilization of professional help within the public healthcare system.

**Methods:**

We conducted a cross-sectional survey with *N* = 1,263 individuals from the general population (mean age 32.56 ± 11.51, 58.2% female) and employed structural equation modeling to investigate associations between stigma (against individuals with psychological disorders and against help-seeking), mental health literacy, MHA usage, and service utilization within the public healthcare system for mental health problems.

**Results:**

MHA usage is high within the general population (40.5% of participants). Results indicate that higher stigma against help-seeking is associated with and increased likelihood of MHA usage, which in turn is positively associated with increased likelihood of service utilization. Symptoms of psychological disorder were associated with higher likelihood of service utilization, but not MHA usage.

**Conclusions:**

It can be concluded that MHA appeal especially to individuals with higher stigma against help-seeking and therefore might provide an opportunity to reach underserviced individuals. At the current time, MHA usage appears to take place mostly in a preventative manner or as a supplement to treatment. Better integration into the public healthcare system might help to exploit both preventative and interventional benefits of MHA.

**Supplementary Information:**

The online version contains supplementary material available at 10.1186/s13690-024-01362-w.

**Table:** 

Text box 1. Contributions to the literature
• Usage of mental health apps (MHA) mostly takes place outside the public healthcare system, therefore risks and benefits are uncertain and potential is most likely not fulfilled.
• Because of their anonymity, MHA might be especially attractive to individuals with high stigma against help-seeking who tend to avoid professional treatment.
• High-quality MHA should be integrated into the public healthcare system reach such underserviced population and encourage help-seeking if indicated.

## Background

Mental health apps (MHA) are digital applications aiming to improve mental wellbeing. This includes intervention-focused apps targeting a wide variety of specific symptoms or mental health conditions (e.g., anxiety, depression, alcohol addiction), as well as apps focused on fostering general wellbeing rather than specific symptomatology through e.g. mindfulness exercises or meditation. With up to 20,000 MHA available for download, the number of available apps is high, but the market is unstructured and volatile [1, 2]. Most apps are also of low quality, not developed by health care professionals, and only a very small proportion provide evidence regarding empirical foundation and effectiveness [3–5]. This low quality might partly explain the high attrition numbers of typically over 90% after 10 days since download [6, 7]. Nevertheless, download-rates are high with for example over 20 million global downloads for apps such as Calm or Headspace, indicating that many people are interested in MHA [3]. This is not surprising considering that individuals with mental health concerns tend to rely more on self-help or informal help rather than on professional treatment, especially when symptom severity is low to moderate [8, 9]. But even in individuals with psychological disorders, the subjectively perceived need for formal treatment is not always given and if so, treatment barriers (e.g. waiting lists) are high [10, 11].

For those MHA that have been empirically validated, research shows promising results. Many studies have shown that MHA can be effective in reducing e.g. symptoms of depression or anxiety and increasing life satisfaction [2, 12, 13]. Even though effect sizes are often small (g = 0.09–0.57 depending on control condition and outcome; [2]), experts agree that especially individuals with low symptom severity or individuals waiting for treatment could benefit from high-quality MHA [14, 15]. MHA could also be of use as a form of preventative care [16] and increase motivation for treatment [17–19].

Since research on usage of self-help in general is rather scarce, little is known on how usage of self-help relates to usage of formal help within the public healthcare system. For MHA as a relatively new form of self-help, some initial reports have been published so far. This preliminary evidence suggests that MHA might encourage help-seeking by reducing stigma and increasing mental health literacy [20, 21]. Self-help resources often present a first step in the help-seeking process and could increase motivation for treatment if necessary [22]. Stigmatizing attitudes against psychological disorders as well as help-seeking present a barrier against formal treatment [23, 24]. Anonymous self-help resources can circumvent this barrier and provide support to individuals with high stigma. MHA as a new form of self-help might therefore be a useful tool to reach individuals with mental health problems who would otherwise not seek professional treatment [19]. If MHA provide psychoeducational content, MHA usage could also be speculated to increase mental health literacy (i.e.; knowledge about mental disorders and their management or prevention [25]). Higher mental health literacy is associated with lower stigma and higher willingness to seek help [23, 25], hinting at the possibility of positive associations between MHA usage, mental health literacy, and seeking professional help for mental health issues. However, the role of MHA within the public healthcare system and the relationship between MHA usage and the utilization of formal help is currently unclear and remains an important question that needs to be addressed.

It could be speculated that MHA usage and service utilization could be positively associated with each other. On the one hand, if MHA usage increases mental health literacy and reduces stigma (as suggested above), improved knowledge of resources and lower stigma might encourage service utilization in the case of mental health problems. If this were the case, this positive association could be exploited to improve care for mental health problems. For example, if high-quality MHA were advertised or provided by health insurance companies, usage might increase and individuals in need of formal treatment might be encouraged to seek help. In a reverse association, if professionals such as general practitioners (GPs) or psychotherapists are consulted for mental health concerns, they might recommend MHA - therefore MHA usage might be increased due to service utilization. If healthcare professionals would recommend usage of high-quality MHA to individuals who might benefit from them, many individuals could be provided with low-barrier support. In fact, it has been suggested to even incorporate validated, effective MHA into public healthcare systems via payment channels similar to medication [26, 27]. The public health system in Germany introduced prescription-based MHA in 2020. However, research reports that only about 1% of the general population were informed about these MHA by their GPs [28], GPs are often skeptic towards MHA (especially in rural areas), and prescription rates are low [29]. This indicates that most MHA usage currently takes place outside the official public health system. Therefore, it is important to explore associations between MHA usage and service utilization to better understand why such potential benefits are maybe not fully exploited at the current time.

This study aims to address this matter by exploring associations between MHA usage and usage of professional treatment in the general population, including possible associations with mental health literacy and stigma. Based on the considerations outlined above, we propose the following:

1. Higher stigma is associated with higher likelihood of MHA usage, because it presents an anonymous form of informal help (as opposed to services within the public healthcare system).
2. Higher mental health literacy is associated with higher likelihood of MHA usage, because a) individuals with higher knowledge of mental health resources are more likely to use them and b) usage might increase mental health literacy.
3. Lower stigma and higher mental health literacy are associated with higher likelihood of utilization of professional help (i.e., services within the public healthcare system).
4. Based on the reasoning behind the proposed associations between mental health literacy and MHA usage, MHA usage might be associated with higher likelihood of utilization of the public healthcare system, because both require knowledge of resources.

## Methods

### Study design, sample and procedure

Data was collected between 09/2022 and 03/2023 via an online survey in the general population in Germany. Participants were recruited via social media, survey platforms, and the crowdsourcing platform Clickworker. Inclusion criteria were age 16 and older, ownership of a smartphone, and sufficient German language skills. *N* = 1,711 participants gave informed consent and participated in the study. After exclusion of participants who immediately dropped out after the sociodemographic questions (*n* = 356), who had technical difficulties filling out the survey (*n* = 41), or who had filled out the survey with obvious lack of diligence (*n* = 51), the final sample size was *N* = 1,263. Due to drop-out during the course of the survey, not all participants completed all measures. Complete data of all relevant measures are available for an analysis sample of *n* = 1,069. Individuals who participated via Clickworker were compensated with 2.77€. The survey procedure was reviewed by the ethics committee of the TU Dresden, Germany (EK306072022), with no ethical objection. A publication with data from the same survey under a different research question (characteristics of MHA users vs. non-users) is currently under review [30].

### Measures

Sociodemographic characteristics (age, gender, education) were assessed via adapted questions from section A of the Diagnostic Expert-System for Psychological Disorders (DIA-X-5) interview [31], which assesses not only diagnostic criteria for psychological disorders, but also sociodemographic aspects and service utilization.

Usage of MHA was assessed via a self-developed questionnaire. Participants gave information on how many different MHA they had used in the past 12 months. For analyses, MHA usage within the last 12 months was coded as a dichotomous variable (yes/no). Participants who had used at least one MHA gave follow-up information on the kind of MHA, the frequency of use, reasons for usage etc. Reasons for usage were selected from a list of possible options, including prescription by GP or psychotherapist.

Utilization of the public healthcare system was assessed via the checklist Q1 of the DIA-X-5 interview [31]. Participants could select services of the public healthcare system they had used in the past 12 months from a list of seven inpatient treatment options (e.g., psychiatric hospital, rehab clinic), six outpatient options (e.g., GP, psychotherapist), and five other options (e.g., counseling). For the purpose of this study, two dichotomous variables were generated to reflect inpatient treatment (yes/no) and outpatient treatment (yes/no) within the last 12 months.

Stigma against individuals with psychological disorders was assessed via the Stigma-9-Questionnaire (STIG-9; [32]). The STIG-9 measures perceived stigma against individuals with psychological disorders via nine items rated on a scale from 0 “disagree” to 3 “agree” and evaluated via the sum score. Reliability was satisfactory with Cronbach’s α = 0.88. Personal stigma against help-seeking was measured with the seven-item version of the Self-Stigma Of Seeking Help Questionnaire (SSOSH; [33]). Items are rated on a scale from 1 “do not agree at all” to 5 “completely agree” and evaluated via the sum score. For this study, the questionnaire was translated into German. To ensure validity, the German items were translated back into English and compared to the original. In case of minor disparities, the German version was adapted for optimal representation of the original item. Reliability was satisfactory with Cronbach’s α = 0.89.

Mental health literacy was measured with two scales of the Multicomponent Mental Health Literacy Measure [34], which were translated into German via the same procedure. In this questionnaire, knowledge about mental health (i.e., about symptoms, treatment, and causes/risk factors of psychological disorders) is assessed with 12 items answered on a scale 1 “do not agree at all” to 5 “completely agree”; and knowledge about resources (i.e., how and where to find support or treatment for mental health issues) is assessed with four items answered on the same scale. Answers are recoded to dichotomous values (0 for values 1 to 3, 1 for values 4 and 5) and each factor is evaluated via the sum score of the dichotomized answers. Reliability for both factors was satisfactory with Cronbach’s α = 0.71 (knowledge) and 0.76 (resources).

In addition to these variables of interest, all participants were screened for possible psychological disorders with the adapted research version of the Composite International Diagnostic Screener (CID-S; [35]). The instrument screens for 19 different psychological disorders via 26 items oriented on the stem screening questions of the DIA-X-5 interview [31] referring to the time frame of the past 12 months. The items are supplemented with questions on impairment and/or treatment in case of an affirmative answer. In the current work, two dichotomous variables (yes/no) were generated: symptoms of a psychological disorder (at least one item affirmed, but no impairment/treatment reported) and impairment (at least one stem question affirmed and additionally either treatment or subjective strain reported). Prior work has shown adequate reliability for the CID-S [35] and stem screening questions of the DIA-X-5 [31].

### Analyses

We employed structural equation modeling (SEM) on complete cases to investigate the associations between MHA usage, service utilization, stigma, and mental health literacy according to our hypotheses. The two factors of mental health literacy were entered as predictors of both measures of stigma. These four variables predicted MHA usage, inpatient treatment, and outpatient treatment. To explore associations between MHA usage and professional treatment, MHA usage also predicted inpatient and outpatient treatment. Symptoms of psychological disorder and positive screening for psychological disorder were also entered as predictors of MHA usage, inpatient treatment, and outpatient treatment.

Because service utilization, stigma, and mental health are known to differ between individuals of different age groups, gender, or education, we explored such associations in our data to identify relevant adjustment variables. Based on this regression-based exploration, prediction of stigma was adjusted for age and education (high school level completed; yes/no), prediction of MHA usage and treatment was adjusted for age, gender, and education. For a visual representation of the full model, see Figure S1 in the supplementary material. Analyses were conducted using the software Stata Version SE17.0 [36].

## Results

### Demographics and descriptive statistics

Sample characteristics and descriptive statistics are displayed in Table 1. With *n* = 511, 40.5% of the participants had used at least one MHA within the last 12 months. Only 11 users (2.2% of users) reported that they had used a MHA because it was prescribed to them by their GP or psychotherapist.

**Table 1:** Descriptive statistics

	**MHA users (*n* = 511)**	**Non-users (*n* = 752)**	**Group comparison**
Age [*M* ± *SD*]	33.00 ± 11.25	32.25 ± 11.69	*F* = 1.32, *p* = .251
*Age group* [*n* (%)]			
16–24	139 (27.2)	216 (28.7)	
25–34	197 (38.6)	308 (41.0)	
35–44	89 (17.4)	116 (15.4)	
45–54	53 (10.4)	52 (6.9)	
55–64	26 (5.1)	48 (6.4)	
65+	7 (1.4)	12 (1.6)	
*Gender* [*n* (%)]			
Female	299 (58.5)	436 (58.0)	χ^2^ = .12, *p* = .940
Male	205 (40.1)	307 (40.8)	
Other	7 (1.4)	9 (1.2)	
*Education* [*n* (%)]			
High school level	371 (78.9)	491 (82.0)	χ^2^ = 1.55, *p* = .213
Below high school level	99 (21.1)	108 (18.0)	
*Mental health literacy* [*M* ± *SD*]			
Knowledge	8.48 ± 2.85	8.77 ± 2.21	*F* = 3.33, *p* = .068
Resources	2.47 ± 1.46	2.47 ± 1.47	*F* = .00, *p* = .965
*Stigma* [*M* ± *SD*]			
Psych. disorders	15.43 ± 5.69	15.58 ± 5.34	*F* = .57, *p* = .449
Self-stigma help-seeking	18.70 ± 3.83	18.28 ± 3.24	*F* = 3.77, *p* = .052
*Service utilization* [*M* ± *SD*]			
Any	**172 (36.0)**	**185 (29.8)**	**χ**^**2**^** = 4.72 *p* = .030**
Inpatient	**52 (10.9)**	**33 (5.3)**	**χ**^**2**^** = 11.72, *p* = .001**
Outpatient	152 (31.8)	178 (28.7)	χ^2^ = 1.26, *p* = .261
*Mental health* [*n* (%)]			
Symptoms of psychological disorder	253 (51.2)	318 (46.8)	χ^2^ = 2.27, *p* = .132
Symptoms and impairment	**165 (33.4)**	**271 (39.9)**	**χ**^**2**^** = 5.10, *p* = .024**

Percentages refer to the respective column. Age is given in years. Mental health literacy = Multicomponent Mental Health Literacy Measure (*MMHLM*). Stigma against individuals with psychological disorders = STIG-9; self-stigma of seeking help = Self-Stigma of Seeking Help (*SSOSH*). Service utilization = DIA-X-5 list Q, coded yes/no. Mental health = CID-5-S, coded yes/no. Available data: education *n* = 1,069; MMHLM *n* = 1,087; stigma *n* = 1,072; service utilization *n* = 1,099; mental health *n* = 1,174

*MHA* Mental health application

MHA users and non-users did not differ regarding sociodemographic characteristics, mental health literacy, or stigma. MHA users showed a lower proportion with symptoms of psychological disorder with impairment (χ2 = 5.10, *p* = 0.024), but more frequently reported having used any treatment options (χ2 = 4.72, *p* = 0.030) and inpatient treatment in particular (χ2 = 11.72, *p* = 0.001).

### Structural equation modeling

**Fig. 1:**
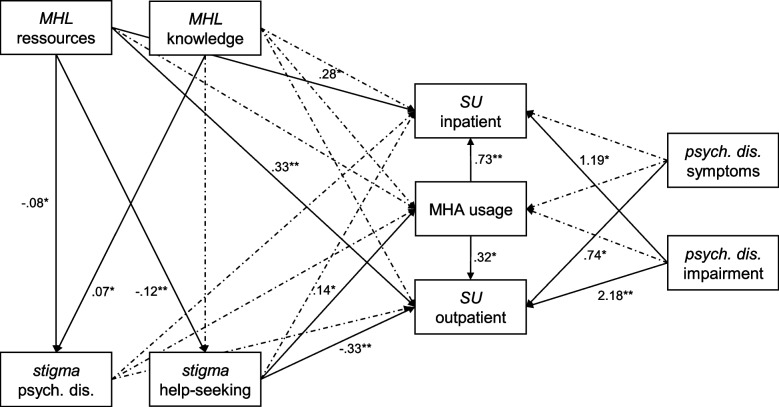
Results of the structural equation modeling. *n* = 1,069. * = significant at α ≤ .05, ** = significant at α ≤ .01. Non-significant paths are shown in grey. MHL = mental health literacy, assessed via the Multicomponent Mental Health Literacy Measure. Stigma against individuals with psychological disorders was assessed via the Stig-9. Stigma against help-seeking was assessed via the SSOSH. MHA = mental health application. Psychological disorder was assessed via the CID-5-S; symptoms = only symptoms affirmed; impairment = symptoms and impairment and/or treatment affirmed. SU = service utilization. Non-dichotomous predictors were standardized

The results of the SEM analysis are depicted in Fig. 1, full tables of the results and the covariances can be found in the supplementary material (Tables S1-S6). Akaike’s information criterion for the model is 9190.873, the Bayesian information criterion is 9424.673. In accordance with the first hypothesis, higher stigma against help-seeking was associated with a higher probability of MHA usage (β = 0.14, *p* = 0.043). Contrary to the second hypothesis, mental health literacy showed no association with usage of MHA.

As predicted in the third hypothesis, higher resource-oriented mental health literacy was associated with a higher likelihood of usage of services within the public healthcare system (inpatient treatment: β = 0.27, *p* = 0.049; outpatient treatment: β = 0.33, *p* < 0.001). Lower stigma against help-seeking was associated with higher probability of service utilization for outpatient treatment (β = -0.33, *p* < 0.001). Knowledge-oriented mental health literacy and stigma against individuals with psychological disorders showed no associations with usage of the public healthcare system. MHA usage was positively associated with service utilization for inpatient (β = 0.73, *p* = 0.003) and outpatient (β = 0.32, *p* = 0.033) treatment.

Symptoms of a psychological disorder and positive screening for psychological disorder were associated with higher probability of service utilization (β = 0.74, *p* = 0.017; β = 1.28, *p* < 0.001), but not with usage of MHA.

## Discussion

The findings of our survey-data from a large convenience sample from the general population reveal that individuals with higher stigma against help-seeking appear to be more likely to use MHA, whereas they are less likely to use professional treatment. Mental health literacy showed no association with MHA usage, but was positively associated with usage of services within the public healthcare system. Interestingly, usage of MHA was associated with a higher likelihood of service utilization. Individuals who reported symptoms of psychological disorders or had a positive screening for psychological disorders were more likely to have sought treatment within the public healthcare system, but not more likely to use MHA. Only a very small number of MHA users reported usage of prescription-based MHA.

These findings are partly in line with previous literature. The association of higher stigma with higher likelihood of MHA usage and lower likelihood of seeking formal treatment underlines the manifold previous research reporting stigma as a barrier against formal help-seeking [23, 24]. It also provides preliminary support for the suggestion that MHA as an anonymous form of self-help might be able to circumvent the barrier of stigma and reach underserviced populations [19]. High stigma against help-seeking might prevent individuals from seeking professional treatment, but not from seeking other forms of anonymous help such as MHA. While it remains uncertain whether MHA usage could actually reduce stigma and encourage help-seeking, it appears possible that MHA present an opportunity to provide support to underserviced populations.

The speculation, however, that MHA usage might be associated with higher mental health literacy could not be supported by our findings. In line with previous research, it appears that mental health literacy is relevant in the context of seeking help within the public healthcare system [23, 25], but not in the context of MHA as self-help. A speculative interpretation of these findings might be that MHA are still too new to be included in a person’s knowledge about sources for help. It might also be considered that individuals with higher mental health literacy do not consider MHA a valid source of support due to the quality issues regarding freely available MHA discussed in the introduction. If this were the case, this might also explain why we did not find a significant association between mental health literacy and MHA usage. Results might therefore differ if MHA become more popular, or if only users of high-quality MHA were included in the study. The idea that - in a reverse association - MHA usage might improve mental health literacy could also no be supported, which is most likely due to the low quality of available MHA and the lack of psychoeducational content in e.g. meditation apps [3–5]. Whether or not MHA usage might be beneficial for mental health literacy if the quality of the MHA is high and psychoeducational content is provided, remains an open question to be examined by future research.

Our findings revealed a positive association between MHA usage and usage of professional treatment. As previous literature has suggested, this could be the case because healthcare professionals recommend self-help resources to supplement treatment [37]. Treatment might therefore increase the likelihood of additional usage of self-help resources such as MHA. On the other hand, research has shown that usage of self-help is very common and often takes place independently from formal help-seeking [8, 38]. It might be the case that individuals first use MHA as self-help, and then seek professional treatment when wellbeing does not improve. It should be taken into account that symptoms of psychological disorders or positive screening for psychological disorders were unrelated to MHA usage, but associated with seeking professional treatment. One might therefore speculate that most of MHA usage might happen for preventative or self-care reasons, not for interventional purposes. This interpretation is supported by a more detailed inspection of the specific apps named by the participants and of the reasons reported for MHA usage, which is discussed elsewhere [30]. Findings centering around “fun” as a reason for usage and self-care apps being more popular than self-help apps, suggest that MHA usage currently takes place not only when symptoms of psychological disorders are experienced, but also for prevention and self-care. Nevertheless, more detailed studies, preferably with longitudinal designs, are needed to further disentangle these associations between mental health, MHA usage, and formal help-seeking.

It is disappointing that only 2.2% of MHA users reported that they had been prescribed a certified MHA. This number is in accordance with previous reports of low prescription rates and skepticism among GPs towards MHA [29]. Despite the recommendations of experts and the processes implemented within the public healthcare system in Germany, integration of self-help via MHA into routine care apparently remains a difficult task. Our findings show that usage of MHA is associated with usage of professional treatment. This indicates that it should be possible for healthcare professionals to ask patients about MHA usage and to recommend or prescribe effective MHA of high quality. Considering that many people use MHA, it appears especially important to ensure that effective MHA of high quality gain more attention in the general public. Through sufficient information of the general population and through training of GPs, prescription-based MHA might gain more popularity and usage of freely available apps of low quality and questionable content might decrease.

Several limitations need to be kept in mind when interpreting our findings. Most importantly, the cross-sectional data cannot establish temporal relations or even causality, all results are merely associations. Longitudinal studies are needed to investigate longitudinal associations between mental health, MHA usage, and service utilization and experimental (interventional) study designs are needed to investigate possible causal relationships. Secondly, our convenience sample is not representative of the general population. Even though we adjusted analyses for age, gender, and education, readers should keep in mind that our sample was rather young and females were overly represented. Since younger individuals and females are for example more likely to seek treatment [39], their overrepresentation in our sample might bias results. Findings from a representative sample including more individuals of higher age-groups and more males might therefore differ from our results due to lower rates of service utilization. Future research should address this. Third, recall and memory might present a problem for measures of MHA usage and service utilization within the past 12 months. Continuous assessment over the course of a year might yield more accurate estimates. Also, we only assessed the kind of service participants had used within the public healthcare system, not the frequency. Limited sessions vs. extensive long-term treatment can therefore not be differentiated in our analyses. Therefore, we cannot infer whether associations between professional treatment and MHA usage differ depending on the intensity of treatment. Lastly, we have no information on the quality of the used MHA. As already mentioned, freely available MHA are often of low quality and lack scientific evaluation regarding their content and their effectiveness. We might therefore assume that the reported MHA usage includes usage of MHA that lack quality. Readers should keep in mind that associations with stigma, mental health literacy, mental health, and even service utilization might differ if only high-quality MHA were used. For example, associations with service utilization might be stronger if MHA provided psychoeducational content on treatment options, and associations with mental health might are likely to be stronger for MHA with content based on cognitive behavioral therapy. This question should also be addressed by future studies.

## Conclusions

In summary, we can conclude that MHA are popular and especially appeal to individuals with higher stigma against help-seeking, therefore providing the opportunity to provide support to people who otherwise might not receive treatment. At the current time, MHA appear to be used primarily in a preventative manner, to support self-care, or to supplement professional treatment. A better integration into the public healthcare system is needed to increase interventional usage (where indicated) and help to provide care to underserviced populations.

### Supplementary Information

Supplementary Material 1

## Data Availability

The dataset and materials analyzed during the study are available from the corresponding author upon request.
