# Supplementary material for: Mental health apps within the healthcare system: associations with stigma and mental health literacy
Source: Arch Public Health. 2024 Aug 16;82:126. doi: 10.1186/s13690-024-01362-w (PMC11328358; doi:10.1186/s13690-024-01362-w)
Supplement: Supplementary file 1 — Supplementary Material 1 [file 13690_2024_1362_MOESM1_ESM.docx]

Fürtjes et al. MHA – Supplementary Material


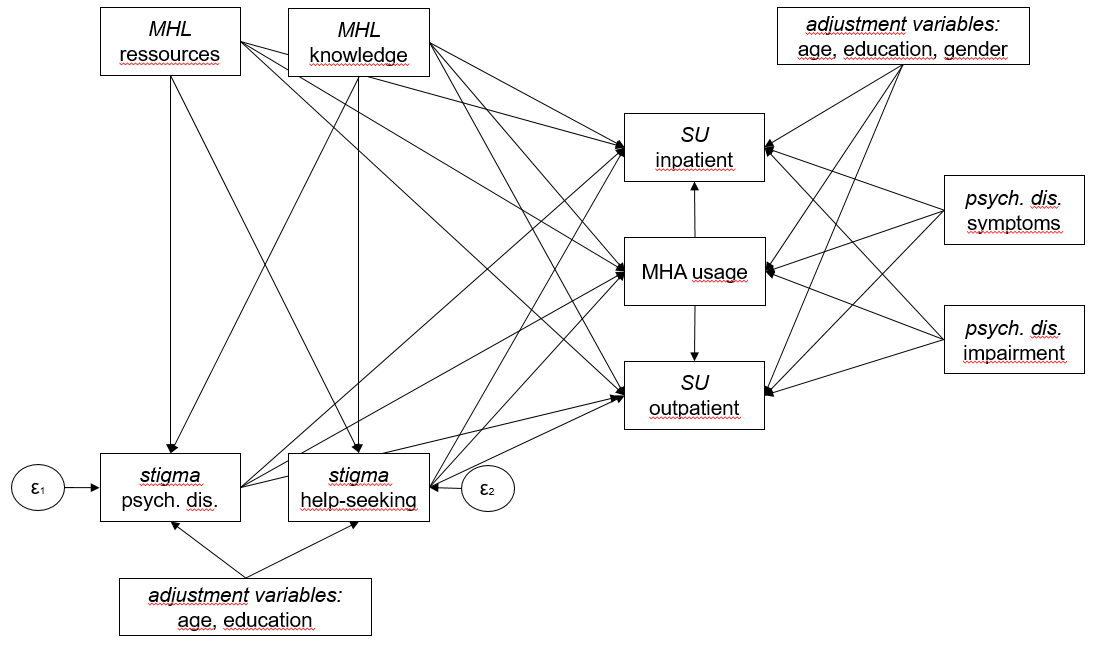


*Figure S1.* Estimated structural equation model. MHL = mental health literacy, assessed via the Multicomponent Mental Health Literacy Measure. Stigma against individuals with psychological disorders was assessed via the Stig-9. Stigma against help-seeking was assessed via the SSOSH. MHA = mental health application. Psychological disorder was assessed via the CID-5-S; symptoms = only symptoms affirmed; impairment = symptoms and impairment and/or treatment affirmed. SU = service utilization.

| *Table S1*. Results of the structural equation modeling | | | | | |
| --- | --- | --- | --- | --- | --- |
| **Outcome** | **Predictor** | **β [*SE*]** | ***p*** | **95%-CI** |  |
| Stig-9 | MMHLM knowledge  MMHLM resources  age  high school level educ. | **.07 [.03]**  **-.08 [.03]**  .00 [.00]  **-.18 [.08]** | **.028**  **.012**  .868  **.018** | **[.01; .14]**  **[-.15; -.02]**  [-.01; .005]  **[-.33; -.03]** |  |
| SSOSH | MMHLM knowledge  MMHLM resources  age  high school level educ. | -.04 [.03]  **-.12 [.03]**  .00 [.00]  -.13 [.08] | .225  **< .001**  .791  .089 | [-.10; .02]  **[-.19; -.06]**  [-.01; .004]  [-.28; .02] |  |
| MHA usage | MMHLM knowledge  MMHLM resources  Stig-9  SSOSH  CID-5-S symptoms  CID-5-S impairment  age  *gender*  female (base)  male  diverse  high school level educ. | -.12 [.07]  .07 [.07]  -.05 [.07]  **.14 [.07]**  .07 [.19]  -.25 [.20]  -.004 [.01]  -.08 [.13]  .01 [.55]  -.21 [.16] | .069  .329  .429  **.041**  .699  .211  .487  .545  .982  .186 | [-.26; .01]  [-.07; .20]  [-.18; .08]  **[.01; .27]**  [-.29; .44]  [-.65; .14]  [-.01; .01]  [-.34; .18]  [-1.07; 1.09]  [-.52; .10] |  |
| SU inpatient | MMHLM knowledge  MMHLM resources  Stig-9  SSOSH  MHA usage  CID-5-S symptoms  CID-5-S impairment  age  *gender*  female (base)  male  diverse  high school level educ. | -.14 [.13]  **.28 [.14]**  .07 [.13]  .01 [.12]  **.73 [.24]**  .28 [.48]  **1.19 [.47]**  **-.02 [.01]**  .08 [.26]  1.02 [.69]  **-.64 [.26]** | .276  **.046**  .603  .930  **.003**  .559  **.011**  **.045**  .759  .143  **.014** | [-.40; .12]  **[.005; .55]**  [-.18; .31]  [-.23; .25]  **[.25; 1.20]**  [-.65; 1.21]  **[.27; 2.11]**  **[-.05; -.001]**  [-.43; .59]  [-.34; 2.37]  **[-1.26; -.13]** |  |
| SU outpatient | MMHLM knowledge  MMHLM resources  Stig-9  SSOSH  MHA usage  CID-5-S symptoms  CID-5-S impairment  age  *gender*  female (base)  male  diverse  high school level educ. | .04 [.09]  **.33 [.08]**  .14 [.08]  **-.33 [.08]**  **.32 [.15]**  **.74 [.31]**  **2.18 [.31]**  -.01 [.01]  **-.44 [.16]**  -.07 [.58]  -.13 [.19] | .670  **< .001**  .067  **< .001**  **.033**  **.017**  **< .001**  .095  **.007**  .898  .497 | [-.13; .21]  **[.16; .49]**  [-.01; .30]  **[-.49; .16]**  **[.03; .62]**  **[.13; 1.35]**  **[1.58; 2.79]**  [-.03; .002]  **[-.75; -.12]**  [-1.20; 1.06]  [-.50; .24] |  |
| *Notes*. Non-dichotomous were standardized. *n* = 1,069. Stig-9 = stigma against individuals with psychological disorders. SSOHS = Self-Stigma of Help-Seeking. MMHLM = Multicomponent Mental Health Literacy Measure. MHA = mental health application (usage coded yes/no). SU = service utilization (coded yes/no). CID-5-S = Composite International Diagnostic Screener. Age is give in years. | | | | | |

| *Table S2.* Covariance matrix for the SEM-equation with Stig-9 as outcome | | | | |
| --- | --- | --- | --- | --- |
|  | MMHLM knowledge | MMHLM resources | age | high school level educ. |
| MMHLM knowledge  MMHLM resources  age  high school level educ. | .001  .000  .000  .000 | .001  .000  .000 | .000  .000 | .006 |
| *Notes.* Stig-9 = stigma against individuals with psychological disorders. SSOHS = Self-Stigma of Help-Seeking. MMHLM = Multicomponent Mental Health Literacy Measure. Age = years. | | | | |

| *Table S3.* Covariance matrix for the SEM-equation with SSOSH as outcome | | | | |
| --- | --- | --- | --- | --- |
|  | MMHLM knowledge | MMHLM resources | age | high school level educ. |
| MMHLM knowledge  MMHLM resources  age  high school level educ. | .001  .000  .000  .000 | .001  .000  .000 | .000  .000 | .006 |
| *Notes.* Stig-9 = stigma against individuals with psychological disorders. SSOHS = Self-Stigma of Help-Seeking. MMHLM = Multicomponent Mental Health Literacy Measure. Age = years. | | | | |

| *Table S4.* Covariance matrix for the SEM-equation with MHA usage as outcome | | | | | | | | | | |
| --- | --- | --- | --- | --- | --- | --- | --- | --- | --- | --- |
|  | MMHLM knowledge | MMHLM resources | Stig-9 | SSOSH | CID-5-S symptoms | CID-5-S impairment | *gender* male | *gender*  diverse | age | high school level educ. |
| MMHLM knowledge  MMHLM resources  Stig-9  SSOSH  CID-5-S symptoms  CID-5-S impairment  *gender* male  *gender* diverse  age  high school level educ. | .005  -.002  .000  .000  -.001  -.002  .001  .000  .000  -.001 | .005  .000  .000  .000  .001  .000  -.001  .000  .000 | .004  -.002  -.002  -.001  .000  .000  .000  .001 | .005  .001  .001  -.001  .000  .000  .000 | .040  .028  .004  -.005  .000  .003 | .035  .003  -.002  .000  .001 | .018  .006  .000  .001 | .304  .000  .001 | .000  .000 | .095 |
| *Notes.* Stig-9 = stigma against individuals with psychological disorders. SSOHS = Self-Stigma of Help-Seeking. MMHLM = Multicomponent Mental Health Literacy Measure. MHA = mental health application (usage coded yes/no). SU = service utilization (coded yes/no). CID-5-S = Composite International Diagnostic Screener. Age = years. | | | | | | | | | | |

| *Table S5.* Covariance matrix for the SEM-equation with SU outpatient as outcome | | | | | | | | | | | |
| --- | --- | --- | --- | --- | --- | --- | --- | --- | --- | --- | --- |
|  | MMHLM know. | MMHLM res. | Stig-9 | SSOSH | CID-5-S symp. | CID-5-S imp. | *gender* male | *gender*  diverse | age | HS level educ. | MHA usage |
| MMHLM knowledge  MMHLM resources  Stig-9  SSOSH  CID-5-S symptoms  CID-5-S impairment  *gender* male  *gender* diverse  age  high school level educ.  MHA usage | .008  -.002  .000  .000  -.001  .000  .001  .000  .000  -.001  .000 | .007  .000  .001  .001  .001  .000  .000  .000  .001  .000 | .006  -.002  -.002  .000  .000  .000  .000  .000  .001 | .007  .001  .001  -.001  -.001  .000  .001  -.001 | .096  .083  .005  -.006  .000  .004  .002 | .096  .004  -.003  .000  .002  -.001 | .026  .009  .000  .002  .000 | .333  .000  .000  .000 | .000  .000  .000 | .036  .002 | .023 |
| *Notes.* Stig-9 = stigma against individuals with psychological disorders. SSOHS = Self-Stigma of Help-Seeking. MMHLM = Multicomponent Mental Health Literacy Measure. MHA = mental health application (usage coded yes/no). SU = service utilization (coded yes/no). CID-5-S = Composite International Diagnostic Screener. Age = years. | | | | | | | | | | | |

| *Table S6.* Covariance matrix for the SEM-equation with SU inpatient as outcome | | | | | | | | | | | |
| --- | --- | --- | --- | --- | --- | --- | --- | --- | --- | --- | --- |
|  | MMHLM know. | MMHLM res. | Stig-9 | SSOSH | CID-5-S symp. | CID-5-S imp. | *gender* male | *gender*  diverse | age | HS level educ. | MHA usage |
| MMHLM knowledge  MMHLM resources  Stig-9  SSOSH  CID-5-S symptoms  CID-5-S impairment  *gender* male  *gender* diverse  age  high school level educ.  MHA usage | .018  -.007  -.001  .000  -.007  -.005  .003  -.002  .000  -.002  .002 | .019  .001  .002  -.001  .001  -.001  .002  .000  .001  -.001 | .016  -.005  -.007  -.004  .001  -.002  .000  .001  .001 | .015  .005  .005  -.005  -.001  .000  .002  -.003 | .219  .189  .019  -.006  .001  .008  .005 | .227  .015  -.001  .000  .004  -.001 | .067  .023  .000  .004  .001 | .481  .000  .002  .004 | .000  .000  .000 | .070  .004 | .059 |
| *Notes.* Stig-9 = stigma against individuals with psychological disorders. SSOHS = Self-Stigma of Help-Seeking. MMHLM = Multicomponent Mental Health Literacy Measure. MHA = mental health application (usage coded yes/no). SU = service utilization (coded yes/no). CID-5-S = Composite International Diagnostic Screener. Age = years. | | | | | | | | | | | |
